# Morphological and electroencephalographic correlates in patients with disorders of consciousness following primary brainstem hemorrhage: a retrospective analysis

**DOI:** 10.3389/fneur.2026.1786548

**Published:** 2026-05-07

**Authors:** Siyuan Ma, Jiawei Shi, Jiajia Liu, Ke Li, Jun Yang, Yingzhun Liang, Jianghong He, Xing Fan, Hui Qiao

**Affiliations:** 1 Beijing Neurosurgical Institute, Capital Medical University, Beijing, China; 2 Department of Neurosurgery, Beijing Tiantan Hospital, Capital Medical University, Beijing, China

**Keywords:** cortical thickness, disorders of consciousness, EEG spectral analysis, primary brainstem hemorrhage, surface-based morphometry

## Abstract

**Background:**

Primary brainstem hemorrhage (PBSH) frequently results in prolonged disorders of consciousness (DOC). Disruption of the ascending reticular activating system (ARAS) is a central pathophysiological mechanism linking brainstem injury to secondary cortical structural and functional changes. In the current study, we employed surface-based morphometry (SBM) and electroencephalography (EEG) spectral analyses to investigate the structure–function interaction in PBSH-induced DOC within the ARAS-mesocircuit framework.

**Methods:**

We retrospectively analyzed the data of 24 PBSH-induced DOC patients and 29 healthy controls. Cortical thickness was assessed via SBM, while EEG spectral powers across different bands were also calculated. Relationships between cortical thickness and EEG spectral metrics in patients with PBSH-induced DOC were explored using a General Linear Model. The correlations between EEG spectral powers and scores on the Chinese version of the Coma Recovery Scale-Revised (CRS-R) were also explored.

**Results:**

Patients with PBSH-induced DOC demonstrated widespread cortical thinning compared to controls, predominantly affecting frontal and temporal regions that constitute primary cortical targets of ARAS projections (*p* < 0.05). Delta and theta power negatively correlated with mean cortical thickness in significant thinning clusters (delta: *ρ* = −0.44, *p* = 0.031; theta: *ρ* = −0.55, *p* = 0.005), with prominent involvement of inferior temporal/fusiform (delta) and superior temporal/postcentral (theta) areas. Higher theta power correlated with lower CRS-R total scores (*ρ* = −0.58, *p* = 0.043), while alpha power showed a similar correlation (*ρ* = −0.61, *p* = 0.020).

**Conclusion:**

This study demonstrates that PBSH-induced DOC is associated with widespread cortical thinning, particularly in ARAS-dependent frontal and temporal regions, and that these structural alterations are reflected in characteristic EEG spectral signatures. These findings offer novel insights into the ARAS-mediated pathophysiological mechanisms underlying PBSH-induced DOC and may inform the development of structure-informed diagnostic and therapeutic biomarkers.

## 1 Introduction

Primary brainstem hemorrhage (PBSH) is recognized as the most devastating subtype of intracerebral hemorrhage, accounting for approximately 5–10% of all intracerebral hemorrhage cases (1, 2). PBSH is characterized by acute onset, rapid progression, and extremely dismal prognosis, with a mortality rate frequently exceeding 50–70% (3, 4). The clinical course and prognosis of PBSH are heavily influenced by hemorrhage location: pontine hemorrhages, which represent the most common subtype, carry the worst prognosis due to their proximity to critical respiratory and cardiovascular centers, whereas midbrain hemorrhages may be associated with somewhat more favorable outcomes. Key prognostic factors include hematoma volume, Glasgow Coma Scale score at admission, and hemorrhage classification (5). Disorders of consciousness (DOC) represent a prevalent clinical manifestation in PBSH patients. Although some patients exhibit early recovery of consciousness after the initial injury, a notable subset will progress to prolonged DOC, which may manifest clinically as unresponsive wakefulness syndrome (UWS)/vegetative state (VS), minimally conscious state (MCS), or persistent coma (6). Enhancing the understanding of PBSH-induced DOC and developing relevant therapeutic interventions will significantly help improve the management of PBSH patients. However, current knowledge remains limited.

As a functional impairment resulting from structural brain damage, PBSH-induced DOC necessarily entails its underlying structural bases. Specifically, the pathophysiological basis of PBSH-induced DOC primarily involves the disruption of the ascending reticular activating system (ARAS), which has extensive projections to cortical regions through both thalamic and extrathalamic pathways (7, 8). Structural damage to ARAS pathways reduces tonic excitatory neuromodulatory input, mediated by monoaminergic and cholinergic projections, to thalamic relay nuclei and cortical regions, representing a disruption of structural connectivity. In turn, this reduction in tonic arousal drive diminishes the physiological synchrony of thalamocortical oscillations and impairs functional network coherence, an effect functionally analogous to, though far more severe than, the reduction in cortical excitability observed during deep sleep (9). While structural disconnection can in principle be assessed via diffusion tensor imaging, the functional consequences, including altered EEG spectral signatures, may serve as dynamic, bedside-accessible surrogates of underlying structural damage.

Such disruptions give rise to widespread thalamocortical and brainstem-cortical dysconnectivity, with functional network perturbations extending beyond the primary lesion site to impact distributed cortical and subcortical regions (10). Of particular relevance to chronic DOC is the occurrence of secondary cortical structural changes remote from the primary injury site. Cortical thinning following ischemic injury is mechanistically attributable to neuronal loss, dendritic arborization reduction, and synaptic pruning driven by energy failure, excitotoxicity, and subsequent apoptotic and neuroinflammatory cascades (11). In the context of PBSH, although the primary hemorrhagic lesion is focal, analogous secondary cortical atrophy can occur through chronic deafferentation, that is, reduced trophic support from severed ascending pathways, and propagating neuroinflammatory processes (12). Previous neuroimaging investigations have demonstrated bilateral cortical thickness reductions in patients with brainstem ischemia, suggesting that similar structural changes may occur in PBSH patients (13).

Electroencephalography (EEG) provides a complementary, high-temporal-resolution window into the functional consequences of these structural disruptions. In DOC populations, well-established EEG markers of impaired consciousness include increases in low-frequency power, reductions in alpha and beta power, and decreases in measures of signal complexity and entropy (14–16). Quantitative EEG parameters have been shown to effectively predict 90-day mortality in PBSH patients, confirming that PBSH is associated with specific electrophysiological changes (17). Notably, the dynamic nature of these structure–function relationships is further underscored by evidence that cortical thickness increases in several regions following recovery from DOC (18), suggesting that EEG spectral signatures and cortical morphology may co-evolve as a function of consciousness level, rather than representing static sequelae of the primary injury.

Given that structural abnormalities form the anatomical basis for functional impairments, it can be inferred that electrophysiological changes may be closely linked to cortical structural alterations in patients with PBSH-induced DOC, and elucidating such structure–function interaction is crucial for developing biomarkers of consciousness assessment and targeted therapeutic interventions. The current study aims to bridge this critical knowledge gap by utilizing surface-based morphometry (SBM) and EEG spectral analyses to systematically investigate the association between alterations in cortical thickness and EEG power spectra in patients with PBSH-induced DOC.

## 2 Methods

### 2.1 Participants and clinical data

We conducted a retrospective analysis of 29 patients diagnosed with chronic DOC secondary to PBSH admitted to the Consciousness Disorders Unit at Beijing Tiantan Hospital between January 2021 and March 2025. The inclusion criteria were as follows: (1) age 18–60 years; (2) first episode of brainstem hemorrhage; (3) duration of DOC exceeding 60 days. Exclusion criteria for all participants included: (1) severe MRI or EEG artifacts or incomplete datasets; (2) significant hydrocephalus or brain deformation; (3) history of neurological or psychiatric disorders; (4) abnormalities in thyroid, hepatic, renal function, or electrolyte balance. Significant hydrocephalus was defined based on clinical diagnosis recorded in medical charts, supported by neuroimaging evidence of ventricular enlargement, as assessed by experienced neuroradiologists. The study was approved by the Ethics Committee of Beijing Tiantan Hospital (KY2022-094-02), and written informed consent was obtained from all participants’ legally authorized representatives in accordance with the ethical guidelines. A control cohort of 29 healthy individuals, matched for age and sex, was selected from the Southwest University Adult Lifespan Dataset (SALD) brain imaging database (19).

### 2.2 Consciousness assessment

Consciousness levels were evaluated using the Chinese version of the Coma Recovery Scale-Revised (CRS-R), comprising six subscales with total scores ranging from 0 to 23 (20). We collected all CRS-R assessments conducted during hospitalization and calculated the mean of at least four evaluations as the final score to ensure measurement reliability.

### 2.3 MRI acquisition and analysis

Structural MRI data were acquired using a 3 T Siemens Magnetom Prisma scanner (Siemens Healthineers, Erlangen, Germany) with a 32-channel head coil. Participants were positioned with foam padding and earplugs to minimize motion artifacts and scanner noise. T1-weighted images were obtained using the following parameters: repetition time (TR) = 1,560 ms, echo time (TE) = 1.69 ms, inversion time (TI) = 778 ms, flip angle = 8°, matrix = 256 × 256, slices = 192, slice thickness = 1.0 mm, voxel size = 1 × 1 × 1 mm^3^. For the SALD control group, T1-weighted images were acquired on a 3 T Siemens Trio scanner with: TR = 1900 ms, TE = 2.52 ms, TI = 900 ms, flip angle = 9°, matrix = 256 × 256, slices = 176, slice thickness = 1.0 mm, voxel size = 1 × 1 × 1 mm^3^.

SBM analysis was carried out using the Computational Anatomy Toolbox (CAT12),^1^ which functions as an extension of the SPM12 software package^2^ on the MATLAB platform. T1-weighted images underwent skull-stripping and registration to the MNI152 standard template via affine transformation. Cortical thickness was estimated using a projection-based thickness method, followed by surface reconstruction and mapping onto the “FsAverage” template to enable inter-subject comparisons. Surface measures were resampled and smoothed with a 15 mm full-width at half-maximum (FWHM) Gaussian kernel. The Desikan–Killiany (DK) atlas was employed to assess regional thickness differences for cortical parcellation. Quality control was implemented using the automated quality assurance protocol CAT12, which categorizes cortical reconstruction quality into grades (e.g., A, B, C, and D, with A representing the highest quality). Subjects with cortical reconstruction quality below grade C would be excluded to ensure the reliability of morphometric analysis.

### 2.4 EEG acquisition and analysis

EEG data were recorded using a standardized 19-channel scalp EEG acquisition protocol based on the international 10–20 system, at a sampling rate of 1,024 Hz, with electrode impedances maintained below five kΩ. During the 30-min recording sessions, participants were seated in a dimly lit, sound-attenuated room and instructed to remain relaxed with their eyes closed. An experienced neurophysiologist monitored all sessions and annotated artifacts in real-time.

EEG data preprocessing and analysis were performed using MNE-Python (version 1.5.0).^3^ Signals were down-sampled to 250 Hz, notch-filtered at 50 Hz to remove power line interference, and bandpass-filtered (1–50 Hz) using a finite impulse response (FIR) filter. Independent component analysis (ICA) was applied to remove electromyographic and electrooculographic artifacts. Following ICA correction, the cleaned continuous recording was visually inspected in 10–30 s scrolling windows. Contaminated data were defined as segments containing residual non-cerebral artifacts, including eye movements or blinks, muscle bursts, gross movements, electrode artifacts, or drifts. The longest continuous 3-min segment with less than 5% residual contamination was selected for spectral analysis. Power spectral density (PSD) was computed using Welch’s method (Fast Fourier Transform length = 256 points, no overlap, mean averaging) across five frequency bands: delta (1–4 Hz), theta (4–8 Hz), alpha (8–12 Hz), beta (12–30 Hz), and gamma (30–50 Hz).

### 2.5 Statistical analysis

Clusters showing relationships between EEG power spectra and cortical thickness were identified using a General Linear Model (GLM), with age, sex, and duration of DOC as covariates. Multiple comparisons were corrected using the cluster-level family-wise error (FWE) method, with a voxel-level threshold of *p* < 0.001 and a corrected cluster-level significance of *p* < 0.05. Clusters with fewer than 100 contiguous triangles were excluded from analysis. Between-group comparisons of cortical thickness in DK atlas regions were performed using the Mann–Whitney *U* test. Spearman’s rank correlation was used to evaluate associations between cortical thickness in significant clusters and EEG power in the DOC group. Statistical significance was set at *p* < 0.05, and multiple comparisons were adjusted using the Bonferroni correction.

## 3 Results

### 3.1 Demographic and clinical characteristics

Following exclusion of individuals with cortical reconstruction quality below grade C, neuroimaging data from 24 patients with DOC and 29 healthy controls were analyzed (Table 1). Five additional patients were excluded due to inadequate cortical surface reconstruction quality (CAT12 grade D), leaving 24 patients for all subsequent analyses. The DOC and control groups were well-matched for age and sex. Within the DOC group, seven patients were diagnosed with UWS and 17 with MCS.

**Table 1:** Demographic and clinical characteristics of study participants.

DOC							HC	
ID	Age (year)	Sex	CRS-R	Diagnosis	TFBI (days)	QC	Age	Sex
1	48	M	6 (0,3,0,1,0,2)	MCS	60	Included	58	F
2	43	M	8 (0,3,2,1,0,2)	MCS	131	Included	54	M
3	53	M	6 (0,3,0,1,0,2)	MCS	157	Included	56	F
4	58	M	3 (0,0,1,1,0,1)	UWS/VS	173	Included	59	M
5	41	M	8 (3,0,2,1,0,2)	MCS	86	Included	56	M
6	41	M	11 (3,3,2,1,0,2)	MCS	130	Included	56	M
7	55	M	7 (1,3,0,1,0,2)	MCS	285	Included	55	M
8	56	M	1 (0,0,0,1,0,0)	UWS/VS	62	Included	41	M
9	51	M	4 (0,0,2,1,0,1)	UWS/VS	275	Included	44	M
10	39	M	5 (0,0,2,1,0,2)	UWS/VS	182	Included	41	M
11	54	M	5 (0,0,2,1,0,2)	UWS/VS	178	Included	35	M
12	46	M	10 (3,3,1,1,0,2)	MCS	120	Included	53	M
13	39	M	6 (0,3,0,1,0,2)	MCS	65	Included	31	M
14	48	M	10 (2,3,2,1,0,2)	MCS	180	Included	56	M
15	59	M	10 (3,2,2,1,0,2)	MCS	333	Included	47	M
16	39	M	2 (0,0,0,1,0,1)	UWS/VS	70	Included	51	M
17	42	M	3 (1,0,0,1,0,1)	UWS/VS	172	Included	38	M
18	41	M	6 (0,3,0,1,0,2)	MCS	145	Included	47	M
19	53	M	9 (1,3,2,1,0,2)	MCS	215	Included	48	M
20	32	M	9 (1,4,2,1,0,1)	MCS	60	Included	39	M
21	49	M	9 (1,3,2,1,0,2)	MCS	262	Excluded	42	M
22	58	F	6 (0,3,0,1,0,2)	MCS	134	Excluded	37	M
23	35	M	11 (3,3,2,1,0,2)	MCS	136	Included	48	M
24	56	F	9 (1,3,2,1,0,2)	MCS	138	Included	49	M
25	47	M	6 (0,1,2,1,0,2)	MCS	153	Included	53	M
26	44	M	8 (1,3,2,1,0,1)	MCS	96	Included	35	M
27	56	M	6 (1,0,2,1,0,2)	UWS/VS	84	Excluded	40	M
28	56	M	5 (0,1,2,1,0,1)	UWS/VS	155	Excluded	58	M
29	38	M	4 (0,0,2,1,0,1)	UWS/VS	190	Excluded	42	M
Age	47.48 (7.82)						47.21 (8.20)	
Sex	27 M/2F						27 M/2F	
CRS-R	6.66 (2.70)						–	
TFBI (days)	152.66 (70.84)						–	

Data are presented as mean (standard deviation) unless otherwise specified. CRS-R scores are presented as total score followed by subscale scores in parentheses (auditory, visual, motor, oromotor/verbal, communication, arousal). The quality control issued by CAT12 with a grade of D is marked for exclusion from the analysis. DOC = disorders of consciousness; HC = healthy controls; MCS = minimally conscious state; UWS/VS = unresponsive wakefulness syndrome/vegetative state; TFBI = time from brain injury; QC = quality control; CRS-R = coma recovery scale-revised.

### 3.2 Cortical thickness alterations in patients with PBSH-induced DOC

**Figure 1:**
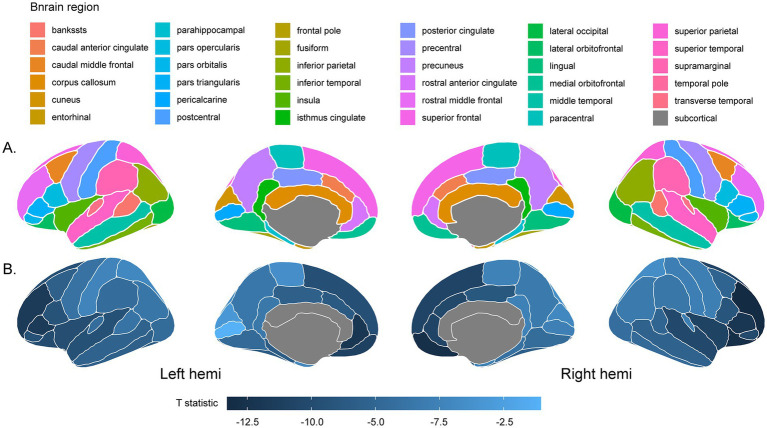
Cortical thickness differences between DOC patients and healthy controls. Brain surface maps showing regions of significant cortical thickness differences between patients with disorders of consciousness (DOC) and healthy controls (HC). **(A)** Desikan–Killiany (DK) atlas parcellation displayed on the “FsAverage” template with distinct colors representing different anatomical regions. **(B)** Statistical t-map highlighting brain regions with significant cortical thickness reductions in DOC patients compared to controls. Cool colors (blue-black) indicate greater thickness reductions in DOC patients. Results are displayed on inflated cortical surfaces for both left and right hemispheres.

Surface-based morphometry revealed widespread cortical thinning in the DOC group (*n* = 24) compared to healthy controls (*n* = 29) across multiple brain regions defined by the Desikan–Killiany atlas (Figure 1). Significant bilateral reductions in cortical thickness were observed in the superior temporal, inferior parietal, precuneus, and rostral anterior cingulate cortices (*p* < 0.05). Additional significant thinning was identified in the left insula and right supramarginal gyrus (*p* < 0.05). Most regions showed significant thinning (e.g., frontal pole: left *t* = −9.36, right *t* = −11.11; lateral orbitofrontal: left *t* = −9.31, right *t* = −11.91; all *p* < 0.05), as detailed in Table 2. By contrast, the cuneus (left hemisphere), pericalcarine cortex (left hemisphere), and superior parietal cortices (bilateral) did not show statistically significant thickness differences after correction, suggesting relative structural preservation of these regions compared to controls.

**Table 2:** Regional cortical thickness comparisons between DOC patients and healthy controls.

Brain region	Hemisphere	Mean DOC (mm)	Mean HC (mm)	T statistic	*p* value	Sig
Bankssts	lh	2.160	2.495	−5.201	0.001	**
	rh	2.260	2.612	−6.936	<0.001	***
Caudal anterior cingulate	lh	1.863	2.401	−7.582	<0.001	***
	rh	1.825	2.422	−8.813	<0.001	***
Caudal middle frontal	lh	2.219	2.626	−7.560	<0.001	***
	rh	2.265	2.620	−7.231	<0.001	***
Cuneus	lh	1.796	1.927	−3.059	0.255	
	rh	1.793	1.999	−4.316	0.007	**
Entorhinal	lh	2.145	2.607	−4.138	0.011	*
	rh	1.922	2.530	−4.841	0.001	**
Frontal pole	lh	2.128	2.785	−9.355	<0.001	***
	rh	2.053	2.739	−11.112	<0.001	***
Fusiform	lh	2.033	2.522	−7.671	<0.001	***
	rh	2.024	2.454	−6.945	<0.001	***
Inferior parietal	lh	2.170	2.495	−4.961	0.002	**
	rh	2.235	2.484	−4.697	0.004	**
Inferior temporal	lh	2.141	2.697	−6.887	<0.001	***
	rh	2.320	2.728	−5.875	<0.001	***
Insula	lh	2.189	3.141	−8.441	<0.001	***
	rh	2.316	3.218	−9.791	<0.001	***
Isthmus cingulate	lh	1.783	2.100	−5.662	<0.001	***
	rh	1.765	2.060	−5.382	<0.001	***
Lateral occipital	lh	1.875	2.211	−5.777	<0.001	***
	rh	1.918	2.229	−5.536	<0.001	***
Lateral orbitofrontal	lh	2.164	2.746	−9.310	<0.001	***
	rh	2.117	2.757	−11.910	<0.001	***
Lingual	lh	1.715	1.991	−6.659	<0.001	***
	rh	1.706	2.002	−7.210	<0.001	***
Medial orbitofrontal	lh	2.031	2.505	−9.973	<0.001	***
	rh	2.009	2.567	−11.341	<0.001	***
Middle temporal	lh	2.230	2.785	−6.431	<0.001	***
	rh	2.437	2.836	−6.884	<0.001	***
Paracentral	lh	2.121	2.303	−3.268	0.153	
	rh	2.125	2.376	−4.068	0.018	*
Parahippocampal	lh	1.268	1.796	−7.517	<0.001	***
	rh	1.368	1.838	−5.571	<0.001	***
Pars opercularis	lh	2.122	2.677	−7.750	<0.001	***
	rh	2.212	2.673	−9.165	<0.001	***
Pars orbitalis	lh	2.176	2.738	−8.420	<0.001	***
	rh	2.200	2.736	−8.568	<0.001	***
Pars triangularis	lh	1.994	2.563	−10.012	<0.001	***
	rh	2.080	2.611	−11.252	<0.001	***
Pericalcarine	lh	1.526	1.599	−1.626	1.000	
	rh	1.523	1.718	−4.994	0.001	***
Postcentral	lh	1.853	2.102	−4.142	0.014	*
	rh	1.891	2.099	−4.334	0.006	**
Posterior cingulate	lh	1.967	2.312	−7.241	<0.001	***
	rh	1.919	2.336	−8.020	<0.001	***
Precentral	lh	2.030	2.345	−5.214	0.001	***
	rh	2.074	2.313	−4.240	0.011	*
Precuneus	lh	2.161	2.392	−5.113	0.001	***
	rh	2.193	2.420	−4.553	0.005	**
Rostral anterior cingulate	lh	2.011	2.703	−10.489	<0.001	***
	rh	2.054	2.700	−9.780	<0.001	***
Rostral middle frontal	lh	2.012	2.509	−10.135	<0.001	***
	rh	1.964	2.512	−11.553	<0.001	***
Superior frontal	lh	2.325	2.809	−9.238	<0.001	***
	rh	2.285	2.844	−9.735	<0.001	***
Superior parietal	lh	2.105	2.295	−3.656	0.057	
	rh	2.123	2.273	−3.106	0.274	
Superior temporal	lh	2.157	2.750	−8.117	<0.001	***
	rh	2.304	2.869	−10.163	<0.001	***
Supramarginal	lh	2.130	2.520	−5.662	<0.001	***
	rh	2.211	2.499	−5.899	<0.001	***
Temporal pole	lh	2.837	3.613	−5.544	<0.001	***
	rh	2.909	3.796	−7.237	<0.001	***
Transverse temporal	lh	1.903	2.270	−6.198	<0.001	***
	rh	1.977	2.333	−5.329	<0.001	***

Regional cortical thickness measurements (in millimeters) for all Desikan–Killiany atlas regions. Statistical comparisons performed using Mann–Whitney *U* tests with Bonferroni correction for multiple comparisons. Significance levels: *p < 0.05, **p < 0.01, ***p < 0.001. lh = left hemisphere; rh = right hemisphere; DOC = disorders of consciousness; HC = healthy controls; *p* value = Bonferroni-adjusted *p*-value.

### 3.3 Associations between EEG power spectra and cortical thickness

**Figure 2:**
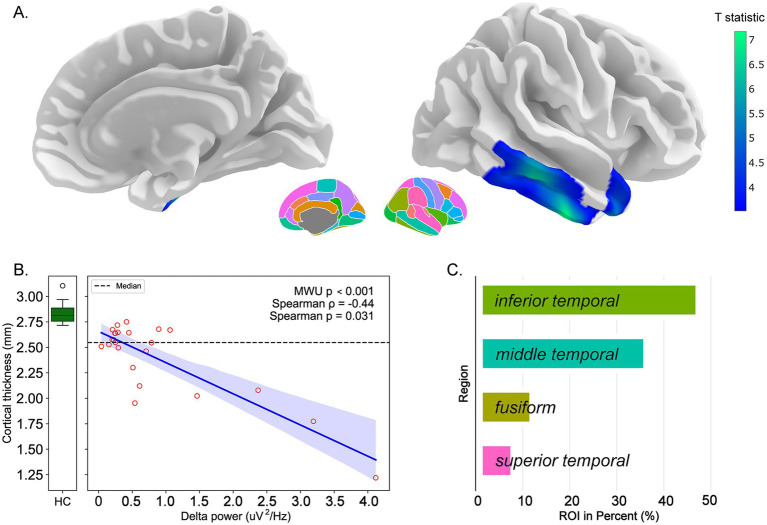
Correlation between delta power and cortical thickness in DOC patients. Structure–function relationships between delta-band EEG power and cortical thickness. **(A)** Three-dimensional rendering of significant surface-based morphometry clusters on the standard brain template, highlighting regions where cortical thickness negatively correlates with delta power (cool colors indicate significant clusters). **(B)** Scatterplot demonstrating the negative correlation between delta power (μV^2^/Hz) and mean cortical thickness (mm) within significant clusters in DOC patients (blue circles). Box plots show median values and interquartile ranges for DOC patients and healthy controls. Statistical comparison: Mann–Whitney *U* test *p* < 0.001; Spearman correlation *ρ* = −0.44, *p* = 0.031. **(C)** Bar chart depicting the percentage contribution of each Desikan–Killiany atlas region to the significant clusters, with inferior temporal and fusiform regions showing the highest representation.

**Figure 3:**
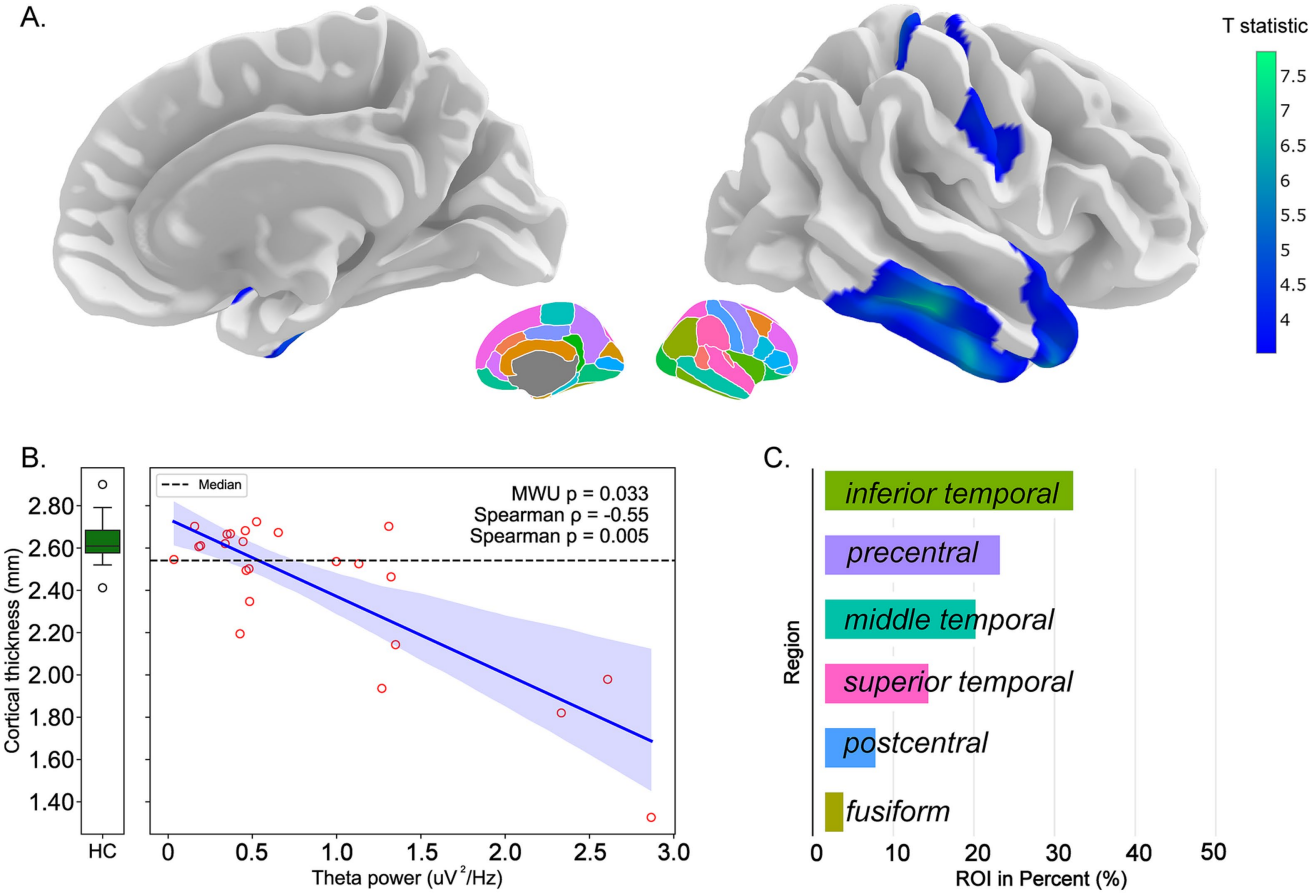
Correlation between theta power and cortical thickness in DOC patients. Structure–function relationships between theta-band EEG power and cortical thickness. **(A)** Three-dimensional rendering of significant surface-based morphometry clusters on the standard brain template, highlighting regions where cortical thickness negatively correlates with theta power (cool colors indicate significant clusters). **(B)** Scatterplot demonstrating the negative correlation between theta power (μV^2^/Hz) and mean cortical thickness (mm) within significant clusters in DOC patients (blue circles). Box plots show median values and interquartile ranges for DOC patients and healthy controls. Statistical comparison: Mann–Whitney *U* test *p* = 0.033; Spearman correlation *ρ* = −0.55, *p* = 0.005. **(C)** Bar chart depicting the percentage contribution of each Desikan–Killiany atlas region to the significant clusters, with superior temporal and postcentral regions showing predominant representation.

Investigation of the relationship between delta and theta power and mean cortical thickness within significant surface-based morphometry clusters revealed significant structure–function associations in the DOC group (Table 3). For delta power (Figure 2), significant clusters exhibiting cortical thinning were identified on the standard brain template. A significant negative correlation emerged between delta power and mean cortical thickness within these clusters (Spearman *ρ* = −0.44, *p* = 0.031), with these regions showing significant thickness reduction compared to healthy controls (Mann–Whitney *U* test, *p* < 0.001). The percentage contribution analysis revealed that the inferior temporal and fusiform gyri showed the highest representation within significant clusters. Similarly, for theta power (Figure 3), significant clusters demonstrated a robust negative correlation with mean cortical thickness (Spearman *ρ* = −0.55, *p* = 0.005) and a considerable reduction compared to controls (Mann–Whitney *U* test, *p* = 0.033). The superior temporal and postcentral regions predominated in the DK atlas percentage distribution for theta-related clusters.

**Table 3:** Statistical summary of structure–function correlation clusters.

EEG bands	Cluster level				Peak level				MNI
	P_FWE-corr_	q_FDR-corr_	K_E_	P_unc_	P_FWE-corr_	T	Z_E_	P_unc_	X,Y,Z
Delta	<0.001	<0.001	1,339	<0.001	0.001	7.17	5.05	<0.001	51 –17 –38
					0.003	6.53	4.77	<0.001	62 –30 –18
					0.011	5.92	4.49	<0.001	47 –34 –22
Theta	<0.001	<0.001	1,404	<0.001	<0.001	7.85	5.31	<0.001	60 –28 –18
					0.006	6.24	4.64	<0.001	55 –15 –34
					0.010	5.98	4.52	<0.001	46 6 –38
	<0.001	0.002	414	<0.001	0.050	5.14	4.09	<0.001	36 –36 61
					0.443	3.89	3.34	<0.001	28 –27 49
					0.642	3.59	3.14	0.001	35 –18 68
	0.018	0.056	222	0.015	0.224	4.32	3.62	<0.001	61 –13 34
					0.224	4.32	3.62	<0.001	64 –7 27
					0.314	4.12	3.49	<0.001	53 –5 25

Statistical parameters for significant clusters identified in the structure–function correlation analysis. P_FWE-corr_ = family-wise error corrected *p*-value controlling for multiple comparisons across the entire brain; q_FDR-corr_ = false discovery rate corrected *p*-value controlling the expected proportion of false positives; K_E_ = cluster extent (number of vertices); P_unc_ = uncorrected *p*-value; T = *t*-statistic value at peak voxel; Z_E_ = equivalent *z*-score; X,Y,Z = Montreal neurological institute (MNI) coordinates in millimeters indicating anatomical location of peak activation.

### 3.4 The correlation of EEG power spectra with clinical consciousness levels

**Figure 4:**
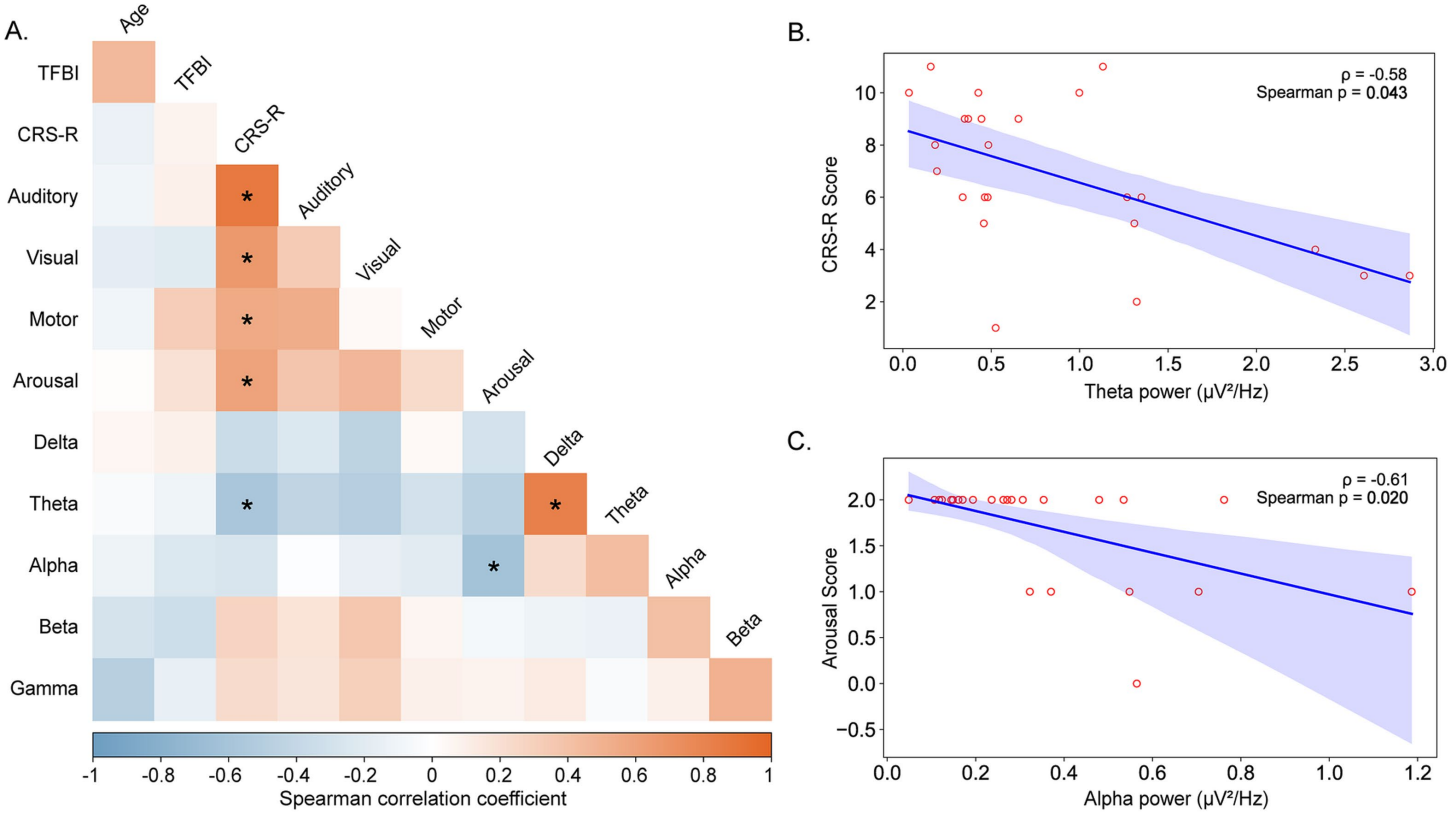
Associations between EEG power spectral density and clinical consciousness measures. Relationships between electroencephalographic power across frequency bands and Coma Recovery Scale-Revised (CRS-R) assessments in DOC patients. **(A)** Heat map displaying Spearman correlation coefficients between CRS-R total and subscale scores (auditory, visual, motor, and arousal functions, respectively) and EEG power across five frequency bands (delta: 1–4 Hz, theta: 4–8 Hz, alpha: 8–12 Hz, beta: 12–30 Hz, gamma: 30–50 Hz). Color intensity represents correlation strength, with red indicating negative correlations and blue indicating positive correlations. Asterisks denote statistical significance: *p < 0.05, **p < 0.01. **(B)** Scatterplot illustrating the significant negative correlation between theta power (μV^2^/Hz) and CRS-R total scores (Spearman *ρ* = −0.58, *p* = 0.043). Each point represents an individual DOC patient. **(C)** Scatterplot showing the significant negative correlation between alpha power (μV^2^/Hz) and CRS-R arousal subscale scores (Spearman *ρ* = −0.61, *p* = 0.020). Trend lines with 95% confidence intervals are displayed for significant correlations.

Analysis of the relationship between EEG power across frequency bands and CRS-R scores in the DOC group (*n* = 24) revealed significant associations (Figure 4). Heat map analysis demonstrated significant negative Spearman correlations between CRS-R scores and power in theta (*ρ* = −0.58, *p* = 0.043) and alpha (*ρ* = −0.61, *p* = 0.020) bands. Specific analysis revealed that higher theta power correlated with lower CRS-R total scores, while increased alpha power was associated with reduced arousal subscale scores.

## 4 Discussion

The current study explored the specific cortical morphological alterations of PBSH-induced DOC and the structure–function interactions under this pathological condition. Moreover, the correlation of EEG power spectra with clinical consciousness levels was also explored.

The observed cortical thinning was most pronounced in frontal and temporal regions, specifically the superior frontal, rostral anterior cingulate, superior temporal, and inferior temporal cortices, which are among the primary cortical targets of ascending ARAS projections via thalamic relay nuclei. These structural alterations can be mechanistically explained within the framework of the “mesocircuit” model, which is a key theoretical construct in neuroscience that is particularly valuable for understanding DOC resulting from severe brain injuries (21, 22). The “mesocircuit” model posits that brainstem lesions significantly impair the functional connectivity among the central thalamus, frontal cortices, and striatum, with damage to the ARAS resulting in deafferentation and decreased metabolic activity within the frontal and temporal cortical regions (22). The ARAS originates in the brainstem and extends upward, playing a crucial role in modulating cortical neuronal activity on consciousness arousal and attention (23). It integrates somatosensory signals to form a diffuse network that directly projects to basal forebrain cortices via the thalamus (24). Due to the substantial dependence of the frontal and temporal cortices on ARAS inputs to sustain physiological function and metabolic homeostasis, brainstem injury may disrupt these critical ascending pathways, leading to a substantial reduction in neuronal activity and subsequent structural atrophy in these cortical regions. Moreover, damage to key subcortical structures, such as the fornix, mammillothalamic tract, and basal forebrain cholinergic pathways, may further exacerbate progressive temporal cortical atrophy (25, 26). These secondary cortical atrophies result from a combination of complex pathophysiological mechanisms that operate across multiple temporal scales, including deafferentation, vascular insufficiency, metabolic dysregulation, and neuroinflammatory processes (27, 28).

In contrast, regions like the cuneus, pericalcarine, and superior parietal cortices exhibit relative preservation, which is likely attributable to their reduced dependence on ARAS inputs. These cortical areas are predominantly involved in visual information processing derived from retinal afferents and exhibit elevated baseline metabolic activity during resting states, thereby enhancing their resistance to deafferentation resulting from brainstem injury (29, 30).

The “mesocircuit” model classifies resting-state EEG power spectra of patients with DOC into four distinct spectral patterns (31–33). Type A features delta-band (0–4 Hz) peaks, indicating complete thalamocortical disconnection with hyperpolarized cortical neurons yielding low-frequency oscillations; Type B shows theta-band (4–8 Hz) dominance, reflecting severe disconnection but partial depolarization with theta bursts; Type C exhibits theta and beta (13–24 Hz) peaks, denoting moderate disconnection with coexisting oscillations; Type D displays alpha (8–13 Hz) and beta peaks, signifying intact networks. In DOC patients, elevated delta and theta power emerge as hallmark electrophysiological signatures of impaired consciousness (14, 15). It may reflect thalamocortical desynchronization and disrupted connectivity between cortical and subcortical areas, leading to sustained hyperpolarization of thalamic neurons and the production of low-frequency oscillations (9, 34). Here we demonstrated that increased delta and theta power correlated significantly with temporal cortical atrophy, which was a pattern echoed in other neurodegenerative conditions, such as Alzheimer’s disease (16), epilepsy (35), aphasia (36), and frontotemporal dementia (37). Structural atrophy in these areas may disrupt the synchronization of thalamocortical oscillations or the modulation of low-frequency oscillations, thereby leading to delta and theta dominance in our cohort. The findings highlight the intrinsic link between structural integrity and functional dynamics in PBSH-induced DOC and indicate that widespread increases in delta-band EEG activity may reflect post-atrophic neural dynamics in the temporal cortex or involve shared pathophysiological mechanisms. In the present study, ‘disorders of consciousness’ and related terms refer to clinically defined states of impaired arousal and awareness, operationalized through the Chinese version of the CRS-R, rather than to any specific philosophical or theoretical framework of consciousness.

The anatomical distribution of cortical thinning is also noteworthy. Rather than broadly claiming that frontal and temporal regions are “important for consciousness” in a generic sense, it is more informative to note that several affected regions overlap with large-scale association cortices and hubs that contribute to arousal-dependent integrative processing, including temporoparietal and midline regions implicated in the default mode network (DMN) (38, 39). In our cohort, thinning involved the precuneus, posterior cingulate, rostral anterior cingulate, and temporoparietal cortices, suggesting that PBSH may have downstream consequences for cortical systems that are especially dependent on intact subcortical arousal regulation. This interpretation does not imply direct measurement of DMN connectivity in the present study, but it provides a plausible network-level context for the observed structural pattern.

At last, our analysis of EEG-clinical correlations further strengthens the utility of electrophysiological markers in assessing consciousness. The robust negative correlation between theta power and CRS-R total scores suggests that theta oscillations are a particularly sensitive marker of global consciousness impairment. This finding aligns with the mesocircuit model, in which theta dominance reflects severe thalamocortical disconnection and partial cortical depolarization (40–42). In addition, the negative association between alpha power and CRS-R total scores is also noteworthy. In DOC patients, residual alpha-band activity may represent disorganized cortical firing patterns (i.e., alpha coma) rather than the coherent thalamocortical oscillations characteristic of routine consciousness. These frequency-specific correlations have important implications for clinical monitoring and prognostication. Theta power emerges as a potential composite biomarker reflecting structural cortical damage and functional consciousness impairment. The dual association of alpha power with structural changes and arousal deficits positions it as a bridge between anatomical disruption and clinical manifestation. This pattern suggests that targeted neuromodulation strategies can benefit from frequency-specific approaches, with theta and alpha bands as primary targets for therapeutic interventions.

Electrophysiological therapeutic modalities, including deep brain stimulation (DBS) (18), spinal cord electrical stimulation (43, 44), transcranial direct current stimulation (45), and transcranial magnetic stimulation (46), have shown promise in ameliorating DOC symptoms. For instance, DBS targeting thalamic structures in DOC patients has been associated with volumetric increases in subcortical regions over 1–7 years post-implantation (18). Although the precise mechanisms remain elusive, modulating subcortical or cortical activity through stimulation may mimic normative thalamic patterns, reverse deafferentation, enhance thalamocortical network synchrony, and facilitate functional and structural reorganization in the post-injury brain, with EEG power spectral features serving as robust metrics for monitoring this recovery process. The observed cortical thinning in temporal and sensorimotor regions has important implications for understanding clinically relevant DOC subgroups. These regions have been shown to distinguish patients in minimally conscious state (47), those with cognitive motor dissociation (48), and those who later recover auditory or language function (49, 50). The degree of temporal cortical preservation may serve as a structural biomarker for predicting recovery trajectories and communication abilities. Future studies could integrate complementary computational approaches, such as model-based effective connectivity (MOU-EC) or whole-brain dynamical modeling (51), with the present morphometric and electrophysiological methods to provide deeper mechanistic insights into the neural processes underlying consciousness recovery in PBSH patients.

Several limitations should be acknowledged. First, the retrospective design and relatively small sample size limited statistical power and prevented subgroup analyses between patients in minimally conscious state and those in unresponsive wakefulness syndrome. Second, the relatively broad age range from 18 to 60 years and potential age-related variability in EEG spectral characteristics may have influenced the results despite statistical adjustment. Third, detailed lesion characteristics, including hematoma location, volume, and classification, were not systematically analyzed, which may affect the extent of ARAS involvement and secondary cortical alterations. Fourth, EEG analysis was based on a single three-minute segment, which may not fully capture temporal fluctuations in patients with disorders of consciousness. Finally, the use of a 19-channel EEG system limited spatial resolution, and the cross-sectional design precludes causal inference. Future longitudinal multimodal studies with more detailed lesion characterization are warranted.

## 5 Conclusion

The present study systematically investigated cortical morphological alterations, structure–function interactions, and EEG-clinical correlations in patients with PBSH-induced DOC. The cohort showed widespread cortical thinning, predominantly in frontal and temporal regions. Moreover, elevated delta and theta power were identified to be negatively correlated with cortical thickness in specific temporal areas. Additionally, theta and alpha powers were significantly associated with clinical consciousness levels. The findings may provide novel insights into understanding PBSH-induced DOC and refining its diagnostic and therapeutic strategies.

## Data Availability

The raw data supporting the conclusions of this article will be made available by the authors, without undue reservation.

## Footnotes

1
http://www.neuro.uni-jena.de/cat/

2
http://www.fil.ion.ucl.ac.uk/spm/software/spm12/

3
https://mne.tools
